# Investigation of a Serine Protease Inhibitor Active in the Infectious Stage of the Human Liver Fluke *Opisthorchis viverrini*

**DOI:** 10.3390/pathogens13080678

**Published:** 2024-08-10

**Authors:** Rosnanee Salang, Wansika Phadungsil, Amornrat Geadkaew-Krenc, Rudi Grams

**Affiliations:** Graduate Program in Biomedical Sciences, Faculty of Allied Health Sciences, Thammasat University, Khlong Luang, Pathum Thani 12120, Thailand; rose.rosnanee@gmail.com (R.S.); wansika_tu@hotmail.com (W.P.); gamornra@tu.ac.th (A.G.-K.)

**Keywords:** *Opisthorchis viverrini*, serine protease inhibitor, serpins, chymotrypsin, kallikrein, thrombin, inhibition, immunoreactivity

## Abstract

Serine protease inhibitors (serpins) participate in the regulation of inflammation, blood coagulation, and complement activation in humans. This research aimed to identify and characterize such inhibitors of the human liver fluke *Opisthorchis viverrini*. Parasite proteins that might contribute to the modulation of host physiology are of particular interest, especially as chronic opisthorchiasis increases the risk of developing biliary cancer. BLAST was used to find hypothetical serpins predicted from the parasite genome data. RNA extraction and reverse transcriptase PCR were used to isolate a serpin cDNA and to determine developmental transcript abundance. The evolutionary relation to other trematode serpins was revealed by phylogenetic analysis. Recombinant serpin was expressed in *Escherichia coli* and used to test the immunoreactivity of human opisthorchiasis sera and the inhibition of human serine proteases. A substantial serpin family with high sequence divergence among the members was found in the genus *Opisthorchis*. A serpin, different from previously analyzed trematode serpins, was cloned. The transcript was only detected in metacercariae and newly excysted juveniles. Human opisthorchiasis sera showed statistically significant reactivity to recombinant serpin. The serpin caused moderate inhibition of thrombin and low inhibition of kallikrein and chymotrypsin. This parasite serpin could be further evaluated as a diagnostic tool for early infection. Kallikrein and thrombin are involved in fibrinolysis; therefore, further research should explore the effects of the parasite serpin on this process.

## 1. Introduction

The liver fluke *Opisthorchis viverrini* is an important human foodborne parasite in the northeastern regions of mainland Southeast Asia. The WHO stated that “foodborne trematode infections cause 2 million life years lost to disability and death worldwide every year” in a fact sheet from 2021 (https://www.who.int/news-room/fact-sheets/detail/foodborne-trematode-infections (accessed on 23 July 2024)). The flukes reside in the host biliary system, and parasite-released embryonated eggs are flushed with the bile into the feces. Following defecation into freshwater resources, the eggs are eventually ingested by freshwater snails in the genus *Bithynia*. Within the snail, the hatched miracidia develop through sporocysts and rediae into cercariae, which, upon release, infest cyprinoid fish and encyst as metacercariae. The consumption of raw/undercooked infested fish then leads to infection when the juveniles excyst in the stomach, reach the duodenum, and migrate into the biliary system (see the life cycle at https://www.cdc.gov/dpdx/opisthorchiasis/ (accessed on 23 July 2024)). Inflammatory responses in the biliary system during heavy infections and the development of cholangiocarcinoma (CCA) in chronic infections have been connected to released and surface-exposed parasite antigens [1,2]. In mammals, serine protease inhibitors are involved in the regulation of biological processes that are also of interest for helminth infection [3]. Inflammation, coagulation, and complement activation are some of the important pathways in which serpins participate. They were found to inhibit a variety of proteases, most often serine proteases, including trypsin, kallikrein, chymase, and plasmin, but some serpins also affect cysteine proteases such as cathepsin. With respect to trematode serpins, the largest body of work is available for blood flukes, including *Schistosoma mansoni*, *S. japonicum*, and *S. haematobium*, dating back several decades. It includes data on molecular characterization, biochemical function, spatial/temporal expression, and biological function in host/parasite interactions. In schistosomes, some serpins have been found to be present in the epithelium of the digestive tract and on the surface of the tegument, while other isoforms had intracellular functions in different tissues, for example, in eggs (reviewed in [4]). Likewise, different serpin genes showed different developmental expression patterns, including high expression in cercariae, indicative of a role in the infection process [5]. They were found to be able to inhibit important host serine proteases, e.g., high-molecular-weight kininogen and elastase.

Recently, several serpins have been analyzed in the cattle/sheep liver fluke *Fasciola hepatica* [6,7], and their possible roles were reviewed in [7]. The importance of distinct isoforms during infection was, comparable to schistosomes, revealed by the upregulation of a serpin prior to the penetration of the host’s intestinal epithelium [8].

In the human liver fluke *Clonorchis sinensis*, which is closely related to *O. viverrini*, three serpins have been described; to avoid confusion, they are referred to here by their UniProt accession numbers E2DHH4, A6YID8, and A0A059VDA6 [9,10,11,12]. The study of transcript abundance showed that E2DHH4 and, in particular, A0A059VDA6 were much more abundant in metacercariae than in mature flukes, while A6YID8 was more abundant in eggs/mature flukes. A6YID8 was localized to eggs and vitelline glands in immunohistochemical analysis and was found in soluble worm extract but not in the excreted/secreted products (ESPs). Recombinant A6YID8 inhibited chymotrypsin but not trypsin, thrombin, elastases, or cathepsin G. In metacercariae, E2DHH4 was localized to the subtegument and oral sucker and A0A059VDA6 was localized to the tegument. Recombinant E2DHH4 inhibited trypsin, chymotrypsin, and thrombin, and recombinant A0A059VDA6 inhibited chymotrypsin. Recombinant E2DHH4 and A0A059VDA6 were detected in immunoblots by sera of (1) rats experimentally infected with *C. sinensis* and (2) rats immunized with ESPs (the developmental stage of ESPs is not mentioned).

In this research, we have isolated and functionally analyzed the first serpin of *O. viverrini* and named it *Ov*SIS (**S**erpin **I**nfectious **S**tage) for its stage-specific expression pattern. Based on its sequence, the selected serpin is different from previously described trematode serpins with a unique RCL sequence. It is evolutionarily close to schistosome SIP but not a direct ortholog of it. This research includes cloning by RT-PCR and sequence analysis, as well as the expression as a recombinant protein in *Escherichia coli* and investigation of human opisthorchiasis sera for reactivity against the *O. viverrini* serpin. Refolded soluble serpin was used in inhibition assays with human serine proteases to determine the inhibition profile. Future research should focus on two major aspects: application as a diagnostic tool for acute infection and the identification of the host and/or parasite serine protease(s) that are the target of this inhibitor.

## 2. Materials and Methods

### 2.1. Parasites

*Opisthorchis viverrini* metacercariae, newly excysted juveniles (NEJs), 2-week-old juveniles, and 6-week-old mature flukes were collected, as previously described [13]. Briefly, metacercariae were obtained from naturally infected cyprinoid fish and either used directly for RNA extraction or to obtain newly excysted juveniles. In addition, the metacercariae were used to experimentally infect Golden Syrian hamsters to obtain 2-week-old juveniles and 6-week-old mature flukes. The ethics approval is found in the Institutional Review Board Statement.

### 2.2. Molecular Cloning of Opisthorchis viverrini SIS cDNA

Metacercariae and juvenile and mature *O. viverrini* were the sources of RNA. RNA isolation was performed using TRIzol reagent (Invitrogen, Carlsbad, CA, USA). Following DNase I (Promega, Madison, WI, USA) treatment, reverse transcription was performed with 100 ng RNA of each sample. RevertAid reverse transcriptase (Thermo Scientific, Vilnius, Lithuania) and an oligo(dT) 18 primer were used. The coding sequence of *O. viverrini* SIS was then amplified from the obtained cDNA by PCR at 50 °C annealing and 72 °C extension temperature. The used primer pair of 5′-gct agc ATG TGT CTC CGC AGT GAG-3′ and 5′-ctc gag GAA AAC TGG TTT GAC CAC-3′ introduced terminal NheI and XhoI restriction endonuclease sites. The primers were designed from a putative serpin-encoding gene and its transcript (Gene: 20320087, GenBank: XM_009171040.1), derived from a whole genome shotgun project of the parasite [14]. The sequence was found through its hypothetical protein (GenPept: XP_009169304.1) by NCBI BLASTP, with the described serpins of *Clonorchis sinensis* [9,10,12] used as query sequences.

Only cDNA produced from metacercarial/NEJ RNA generated a PCR product. It was inserted into the pGEM^®^-T Easy vector (Promega, Madison, WI, USA) using the introduced NheI and XhoI restriction endonuclease sites, and the DNA sequence of the insert was verified by conventional Sanger DNA sequencing (Macrogen, Seoul, Republic of Korea). The analyses, retrieval, and editing of sequences were performed using EMBOSS 6.6 [15], Muscle 5 [16], SignalP 6 [17], BLAST services (https://blast.ncbi.nlm.nih.gov/Blast.cgi (accessed on 18 June 2024)), the UniProt database (https://www.uniprot.org (accessed on 25 June 2024)), and the NCBI nucleotide database (https://www.ncbi.nlm.nih.gov/nucleotide (accessed on 18 June 2024)).

### 2.3. Expression and Purification of Recombinant OvSIS

The cDNA insert encoding *Ov*SIS was released from the pGEM^®^-T Easy vector using the introduced NheI and XhoI restriction endonuclease sites and inserted into the expression vector pET21b (Novagen, Darmstadt, Germany) by ligation. The cloning procedure led to the addition of amino acids LEHHHHHH at the C-terminus of recombinant *Ov*SIS, and the inserted sequence was verified again by sequencing, as detailed above. *Escherichia coli* BL-21(DE3) pLysS was used as the expression host. Following chemical transformation, single positive colonies were identified by using colony PCR and restriction endonuclease digestion. Single positive colonies were inoculated in 5 mL of Luria-Bertani (LB) medium supplemented with ampicillin (100 µg/mL), chloramphenicol (34 µg/mL), kanamycin (12.5 µg/mL), and tetracycline (3.125 µg/mL). The cultures were incubated by shaking at 37 °C overnight. Briefly, 5 mL of starter culture was added to 100 mL of fresh medium with antibiotics and incubated at 37 °C and 250 rpm until the OD600 reached ~0.5–0.6. Then, 1 mM isopropyl-β-D-1-thiogalactopyranoside (Fermentas, Vilnius, Lithuania) was used to induce the expression of recombinant *Ov*SIS. Four hours after induction, the bacterial cells were harvested by centrifugation at 6000× *g* and 4 °C for 30 min. The recombinant protein was purified by Ni-NTA affinity chromatography (QIAGEN, Hilden, Germany) under denaturing conditions following the manufacturer’s instructions. The purified protein was refolded by stepwise dialysis in urea to obtain soluble and active serpin. In detail, 1 mL of eluate was transferred into a dialysis bag (Spectra/Por 3 RC Dialysis Tubing, MWCO 3.5 kD, Spectrum Laboratories, Repligen, Rancho Dominguez, CA, USA), and the closed bag was placed in 1000 mL of 6 M urea, 20 mM Tris-HCl (pH 8.0), and 0.1 mM DTT and incubated with slow stirring at 4 °C for 3 h. This step was repeated and then followed by two incubation steps each at 4 and 2 M urea at 4 °C for 3 h. Afterwards, the protein solution was incubated in 20 mM Tris-HCl (pH 8.0) at 4 °C for 3 h. Following a final change in the buffer, the incubation was continued overnight at 4°C. The protein solution was centrifuged at 5000× *g* and 4 °C for 10 min, and the supernatant was stored at −20 °C. No precipitates were observed during dialysis and the subsequent freeze/thaw steps when the refolded protein was used in further experiments. The protein concentration was determined by a Bradford assay (Bio-Rad Laboratories, Hercules, CA, USA) and showed that 2.24 mg r*Ov*SIS was obtained. Standard 12.5% SDS-PAGE in 0.025 M Tris-HCl (pH 8.9), 0.2 M glycine, and 0.1% (*w*/*v*) SDS running buffer was used to analyze the purity of the refolded protein. The samples (pre- and 4-h-induced bacterial protein equivalent to ~80 µL culture volume, 1 µg refolded r*Ov*SIS) were mixed at a ratio of 1:1 with 0.125 M Tris-HCl (pH 6.8), 0.2 M DTT, 0.02% (*w*/*v*) bromophenol blue, 4% (*w*/*v*) and SDS, 20% (*v*/*v*) sterile glycerol, heated at 95 °C for 5 min, cooled on ice for 1 min, and then added to the wells. The Broad Range Molecular Weight Standards (Bio-Rad Laboratories, Hercules, CA, USA) were run in parallel at an amount of ~10 µg and used to calculate the molecular weight of r*Ov*SIS. The proteins were separated at 20 mA constant current until the front dye had moved to the bottom of the gel. The separating gel was washed in distilled water, submerged in staining solution (0.05% Coomassie Blue G-250, 35 mM HCl), heated in a microwave oven for 20 s, and incubated with gentle shaking overnight. The gel was destained in distilled water, and an image was captured on a flatbed scanner (CanoScan LiDE 700F, Canon, Bangkok, Thailand).

### 2.4. Reverse Transcriptase PCR Analysis

Total RNA was extracted from NEJs, 2-week juveniles, and 6-week mature *O. viverrini* and treated with DNase. Briefly, 200 ng of RNA from each stage were used for reverse transcription with RevertAid reverse transcriptase (Thermo Scientific, Vilnius, Lithuania) and the gene-specific reverse primer 5′-CATGCATCATTGGGACATTC-3′. Conventional PCR was then performed under the conditions described in Section 2.2 with the gene-specific forward primer 5′-AGTCGCACTCCCTTAGATTG-3′ and the above-mentioned reverse primer to produce a 640 bp PCR product. *O. viverrini* actin (*Ov*Actin) was used as an internal control and amplified with primers 5′-TCGAGAGAGATGACACAGA-3′ and 5′-GATATCACGCACGATTTCTC-3′ to produce a 292 bp product. The RT-PCR products were resolved on a 1% agarose gel.

### 2.5. Phylogenetic Analysis of Serpins in the Genera Opisthorchis/Clonorchis and Schistosoma

Forty-six full-length trematode serpin sequences were fetched from UniProt (https://www.uniprot.org (accessed on 8 June 2024)). They included ten sequences from *O. felineus*, seven sequences from *O. viverrini,* nine sequences from *C. sinensis*, six from *S. haematobium*, five from *S. japonicum*, and nine sequences from *S. mansoni*. The sequences were aligned in Muscle v5 [16]. Trimal v1.4 [18] was used with the automated1 option to prepare the aligned sequences for phylogenetic analysis. PhyML v3.3.20220408 [19] was used for analysis under the LG model, with a starting BioNJ tree and Bayes support.

### 2.6. Indirect ELISA of Human Opisthorchiasis Sera with Recombinant OvSIS

Indirect ELISA was used to determine the reactivity of human opisthorchiasis sera against recombinant *Ov*SIS (r*Ov*SIS). The human sera used in this study were collected in Thailand and were classified as normal or infected based on the microscopic investigation of stool samples for parasite eggs [20]. First, 100 ng of r*Ov*SIS I2 in 100 µL coating buffer (30 mM Na_2_CO_3_ and 75 mM NaHCO_3_) were transferred to the wells of a 96-well microtiter plate (NUNC, Roskilde, Denmark) and incubated at 37 °C overnight. The next day, the wells were washed with 10 mM PBS (pH 7.2) and 0.05% Tween 20. Non-specific binding was blocked with a 0.25% BSA blocking solution. Thirty human opisthorchiasis sera were randomly assigned to three groups of 10 samples each, and a group of ten normal human sera was used as a negative control. At first, the sera (diluted 1:100) were tested as pooled sera (*n* = 10), and when found to be reactive with r*Ov*SIS, they were tested individually. Each of the forty serum samples was diluted to 1:100 and added to a well. The plate was incubated at 37 °C for 1 h. Polyclonal rabbit anti-human IgG HRP conjugated antibody (Dako, Glostrup, Denmark) at 1:6000 dilution was added as a secondary antibody and incubated at 37 °C for 1 h. The substrate o-phenylenediamine dihydrochloride (OPD, Sigma-Aldrich, St. Louis, MO, USA) was used for colorimetric detection. The reaction was stopped with 3 M H_2_SO_4_ solution, and absorption was measured at 492 nm on a plate reader (VarioSkan Flash, Thermo Scientific, Waltham, MA, USA). The assay was performed in duplicate. Statistical analysis of the data by an unpaired *t*-test was performed in Prism version 10 (GraphPad Software, LLC, Boston, MA, USA). The ethics approval is found in the Institutional Review Board Statement.

### 2.7. Inhibition of Human Serine Proteases by rOvSIS

The inhibition properties of r*Ov*SIS were tested with native human serine proteases. The residual proteolytic activity was determined after incubating the serine proteases with r*Ov*SIS in the presence of protease-specific fluorogenic substrates in black flat-bottom microtiter plates (Thermo Scientific, Roskilde, Denmark). Details of the used protease and substrate concentrations and buffer composition are shown in Table 1. Sources of proteases and substrates: chymotrypsin from the human pancreas (C8946, Sigma-Aldrich, St. Louis, MO, USA) with substrate Suc-Ala-Ala-Pro-Phe-MCA (3114-v; Peptide Institute Inc, Ibaraki-Shi, Japan), thrombin from human plasma (T6884, Sigma-Aldrich, St. Louis, MO, USA) with substrate Boc-Val-Pro-Arg MCA (B9385, Sigma-Aldrich, St. Louis, MO, USA), and kallikrein from human plasma (K2638, Sigma-Aldrich, St. Louis, MO, USA) with substrate Z-Phe-Arg-MCA (C9521, Sigma-Aldrich, St. Louis, MO, USA). Each serine protease was incubated with 0.5 µM r*Ov*SIS in 100 µL of buffer at 37 °C for 30 min. Then, 100 µL substrate solution was added, and enzyme activity was recorded for 30 min. PMSF at a concentration of 20 mM was used as a positive control inhibitor. Measurements were carried out in duplicate, and the experiment was repeated twice. The residual enzyme activity was measured by monitoring the release of fluorescence with excitation at 355 nm and emission at 460 nm on a Varioskan Flash spectral scanning multimode reader (Thermo Scientific, Waltham, MA, USA). Statistical analysis and graphical representation of the data were performed in Prism version 10 (GraphPad Software, LLC, Boston, MA, USA).

## 3. Results

### 3.1. Molecular Cloning of OvSIS and Analysis of Sequence and Structure

This is the first analyzed serine protease inhibitor (serpin) of *Opisthorchis viverrini*. The sequences of hypothetical serpins of *O. viverrini* were identified by using NCBI BLASTP to search the relevant databases at NCBI GenBank. A total of 48 sequences predicted from the genome data of *O. viverrini* (BioProject: PRJNA259756 [14]; BioProject: PRJNA230518, Mitreva et al., unpublished) were found. From this set of hypothetical serpins, XP_009169304 (UniProt: A0A075AEP2) was selected for analysis because (1) the sequence was complete, and (2) it was highly different in its sequence to previously analyzed serpins in the closely related trematode *Clonorchis sinensis* [9,10,12]. In support of this predicted protein, highly conserved orthologous serpins were found by BLAST searches in *C. sinensis* (UniProt: A0A419PW56) and *O. felineus* (UniProt: A0A4S2MGE0), as shown in Table 2. The *O. felineus* sequence originates from a transcriptome project and, thereby, supports the conclusion that this protein is indeed produced. No developmental stage was specified for the *O. felineus* transcript.

Total RNA was isolated from various developmental stages (metacercariae, newly excysted juveniles, juveniles, and mature parasites) of *O. viverrini* and used for reverse transcriptase PCR with a gene-specific primer pair to obtain the coding sequence of *O. viverrini* A0A075AEP2. A PCR product was obtained from metacercarial and NEJ-stage RNA, and the sequencing demonstrated that the correct 1212 bp sequence from the start codon to the stop codon had been amplified. The deduced 403-residue amino acid sequence had a calculated molecular weight of 46 kDa and did not contain a signal peptide. Due to the finding that the transcript was present in the human infectious stage but not in later stages, the protein was termed *O. viverrini* Serpin Infectious Stage (SIS, *Ov*SIS). The nucleotide sequence of *Ov*SIS is available in the EMBL, GenBank, and DDJB databases under the accession number PP968418.

**Figure 1:**
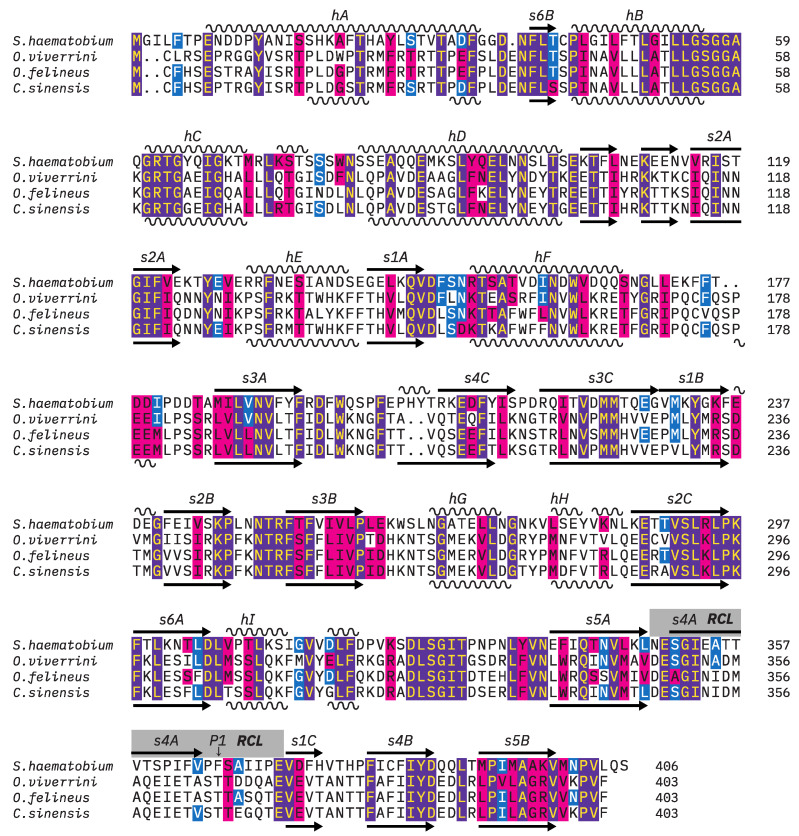
Multiple sequence alignment of *S. haematobium* SPI (UniProt: Q26502), *O. viverrini* SIS (UniProt: A0A075AEP2), *C. sinensis* SIS (UniProt: A0A419PW56), and *O. felineus* SIS (UniProt: A0A4S2MGE0). The α-helical and β-strand regions of *Sh*SPI and *Ov*SIS based on their AlphaFold native state models [23] are indicated at the top and bottom, respectively. The numbering of the secondary structures follows α_1_-antitrypsin [24]. The region of the reactive center loop (RCL) with the P1 residue is indicated by a gray bar. Strand 4A in the RCL was manually added as it forms and integrates into β-sheet A after cleavage at P1. Sequence conservation using the BLOSUM 62 similarity matrix and *Sh*SPI as the reference sequence is indicated by color shading (fully conserved: purple background, ≥50% conserved: blue background, and similar: red background). The graphical representation of the aligned sequences was created using TEXshade v1.28 [25].

Further BLAST searches for homologous proteins in more distantly related trematode species resulted in matches to a hypothetical *Fasciola hepatica* serpin (UniProt: A0A4E0RBU2, 35% identity, 56.7% similarity), schistosome SPI (serine protease inhibitor), *S. mansoni* SPI (G4LYU6, 32.8% identity, 56.0% similarity), *S. japonicum* SPI (C1LNS7, 34.0% identity, 54% similarity), and *S. haematobium* SPI (UniProt: Q26502, 31.8% identity, 53.3% similarity). Using BLAST to compare *Ov*SIS to human serpins, it was found that those clustered into sub-family B showed higher sequence similarity than those of sub-family A, e.g., the human leukocyte elastase inhibitor, i.e., LEI (UniProt: P30740, 21.5% identity, 37.6% similarity, 10.6% gaps). This does not imply that these serpins have equivalent biological function(s) in humans and trematodes. Human serpin B members as well as all discussed trematode serpins lack a signal peptide. Schistosome SPI was selected for comparison to *Ov*SIS because it is, by sequence, the closest described trematode serpin and its structure has already been analyzed [21,22]. A multiple alignment of *Sh*SPI and *Opisthorchis*/*Clonorchis* SIS is shown in Figure 1.

**Figure 2:**
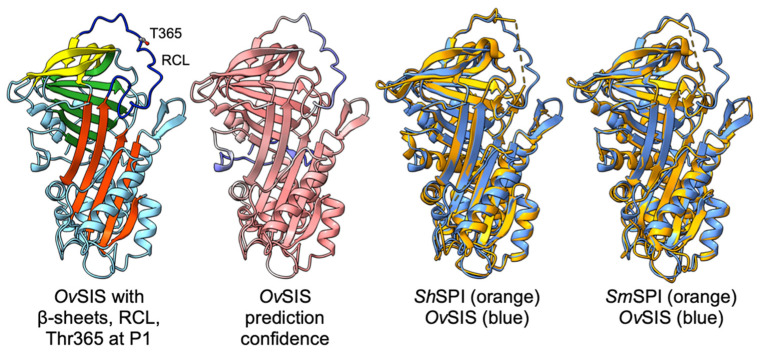
*Ov*SIS structure model predicted in AlphaFold [23] and obtained from the AlphaFold Protein Structure Database [26]. The *Ov*SIS model with the three β-sheets A (red), B (green), and C (yellow), RCL (dark blue), and threonine 365 in the P1 position is shown on the left. The *Ov*SIS model prediction confidence from high to low is indicated by red to blue shading, respectively. Overlays with *Sh*SPI (PDB: 3STO) and *Sm*SPI (PDB: 6SSV) demonstrate structural conservation between the three serpins. Graphical representations were created using ChimeraX [27].

The multiple alignment (Figure 1) shows that the overall secondary structure, with its α-helical and β-strand content, is conserved between the parasite serpins. This is also evident in the tertiary structure model of *Ov*SIS compared to the experimentally resolved structures of *S. haematobium* and *S. mansoni* SPI [21,22] (Figure 2). The extended helix D of schistosome SPI [20] is also found in SIS. The AlphaFold prediction confidence values of *Ov*SIS were low at the N-terminal end up to F32 and in the reactive center loop (RCL), as shown in Figure 2.

### 3.2. Phylogenetic Analysis of Serpins in the Genera Opisthorchis/Clonorchis and Schistosoma

**Figure 3:**
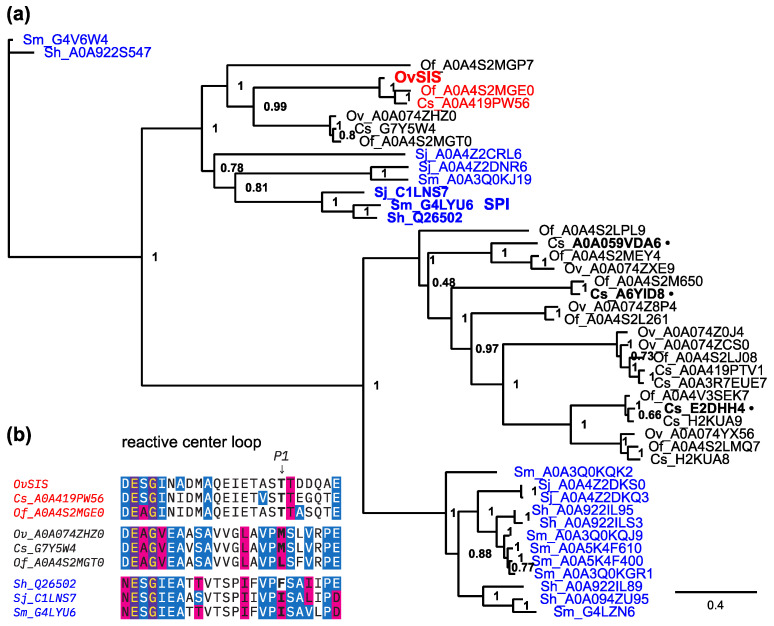
(**a**) Phylogenetic tree of trematode serpins specified by their UniProt accession numbers. The three previously described serpins of *C. sinensis* are indicated with a trailing •. Schistosome serpins are shown in blue, with the SPI in bold lettering. *Ov*SIS and its orthologs are shown in red. *Opisthorchis*/*Clonorchis* SIS is not orthologous to schistosome SIP. (**b**) Sequence conservation of the reactive center loop in evolutionarily close serpins (specified by their UniProt accession numbers) in the species *O. viverrini* (Ov), *O. felineus* (Of), *C. sinensis* (Cs), *S. haematobium* (Sh), *S. japonicum* (Sj), and *S. mansoni* (Sm). The P1 position in the protease interaction is indicated. The color shading indicates fully conserved: purple background, ≥50% conserved: blue background, and similar: red background.

As mentioned before, *Ov*SIS was selected based on its high sequence divergence from the three previously reported *C. sinensis* serpins. These serpins show similar low sequence conservation with *Ov*SIS, as does human LEI at around 19–22% identity and 37–40% similarity. Indeed, the phylogenetic tree calculation placed all analyzed *C. sinensis* serpins on a different branch from *Ov*SIS (Figure 3a). *Ov*SIS and the orthologous *C. sinensis* and *O. felineus* SIS are more closely related but not orthologous to schistosome SPI. The SPI orthologs are potentially *O. viverrini* A0A074ZHZ0, *C. sinensis* G7Y5W4, and *O. felineus* A0A4S2MGT0, as can be seen in the constructed tree and the sequence alignment of their reactive center loop in Figure 3a,b.

### 3.3. Developmental Expression of OvSIS RNA

**Figure 4:**
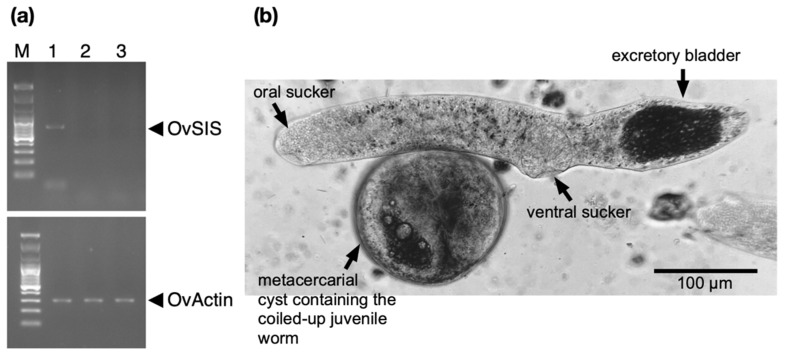
(**a**) RT-PCR products resolved in 0.8% agarose gels. M: marker, 1: newly excysted juveniles, 2: 2-week-old juveniles, and 3: mature parasites. *O. viverrini* β-actin (292 bp) was used as a positive control. A 640 bp *Ov*SIS cDNA was only produced with RNA of newly excysted juveniles. (**b**) Micrograph of a newly excysted *O. viverrini* juvenile (top) and a metacercaria (bottom), the developmental stages in which *Ov*SIS is present. The dark granule-filled excretory bladder, which can also be seen through the cyst wall, is the most prominent. Other outstanding morphological features are the oral and ventral suckers. It is thought that parasite-secreted serine proteases participate in the release of the encysted juveniles and that cognate serpins regulate their excess activity.

Transcripts of the *Ov*SIS gene (NCBI GenBank Gene: 20320087) were detected by RT-PCR in newly excysted juveniles but not in later juveniles or mature flukes (Figure 4a). Originally, we had cloned the *Ov*SIS cDNA from metacercarial RNA. This suggests that the protein is already produced in metacercariae, and the metacercarial presence of RNA has also been reported in previous analyses of serpins in *C. sinensis*. All three analyzed *C. sinensis* serpins showed a wider expression range at the RNA level than *Ov*SIS up to the mature stage but, interestingly, the amounts of RNA of A0A059VDA6 and E2DHH4 were much higher in metacercariae than in the mature fluke [10,12]. It can be assumed that these serpins are required in large amounts during the infection process. Whether they have a redundant function or if they prefer unique serine proteases as targets remains to be determined. Metacercariae and newly excysted juveniles represent the infectious stage of the parasite for its human host. As shown in Figure 4b, these developmental stages are of microscopic size. The excretory bladder is filled with dark pigment granules. In the lung fluke *Paragonimus westermani*, cysteine proteases were located on these granules and speculated to be released for excystment [28].

### 3.4. Analysis of Human Opisthorchiasis Sera Against Recombinant OvSIS

**Figure 5:**
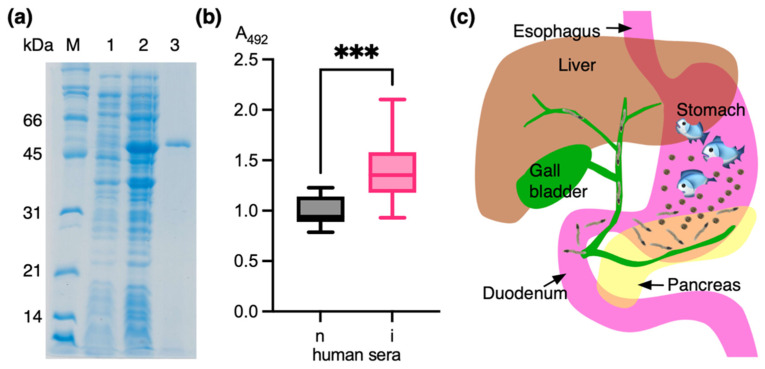
(**a**) SDS-PAGE showing pre-induced (1) and IPTG-induced bacterial proteins (2) and affinity-purified recombinant *Ov*SIS (3). The protein migrates at the expected 46 kDa molecular weight. (**b**) Absorption values of two groups of human sera against recombinant *Ov*SIS obtained by indirect ELISA. n: normal sera (*n* = 10). i: sera of *O. viverrini*-infected individuals (*n* = 30). The minimum, 25th percentile, median, 75th percentile, and maximum are indicated in the box plots. The star symbols indicate that all infected sera were statistically significantly different (*** *p* < 0.05) from the normal sera. (**c**) Human infection with *O. viverrini*. The metacercariae are released from infested undercooked fish in the stomach. The acidic environment activates the excystment of the juveniles. The newly excysted juveniles are released with the stomach content into the duodenum and migrate via the sphincter of Oddi into the biliary system (green).

Recombinant *Ov*SIS was expressed as an insoluble protein in *E. coli*. After purification of the urea-solubilized protein by Ni-NTA affinity chromatography (Figure 5a), it was refolded by stepwise dialysis and tested with the immune sera of thirty individuals naturally infected with the parasite; a positive reaction was observed (Figure 5b). An unpaired *t*-test between normal and infected sera showed a statistically significant difference with a *p*-value of 0.0003. Mean ± standard deviation absorbance values of normal and infected sera were 0.9900 ± 0.1474 and 1.386 ± 0.2990. As schematically shown in Figure 5c, the metacercariae are released when infested fish is digested in the stomach. Gastric juices then stimulate excystation, and once released from the stomach, the newly excysted juveniles (NEJs) must migrate to the sphincter of Oddi/ampulla of Vater to reach the biliary system. Therefore, *Ov*SIS must be released from the start of excystation and stimulate an immune response in the contacted areas of the gastrointestinal tract and biliary system for several days before gene expression is stopped.

### 3.5. Inhibition of Human Serine Proteases by Recombinant OvSIS

**Figure 6:**
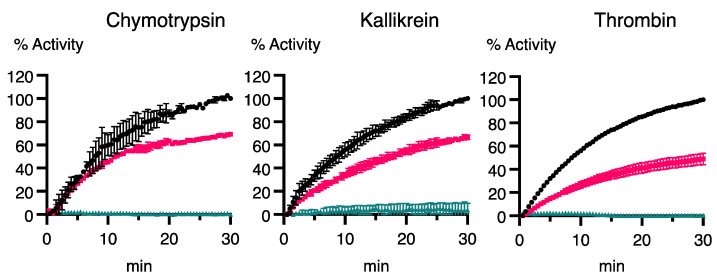
Inhibition kinetics of *Ov*SIS against human serine proteases. Proteases without inhibitors are indicated in black, proteases incubated with recombinant *Ov*SIS are shown in red, and proteases incubated with 20 mM PMSF are shown in teal. After 30 min of preincubation with 0.5 µM recombinant *Ov*SIS and 30 min of incubation with the substrate, the activities of 1 nM chymotrypsin, 1 nM kallikrein, and 0.1 U thrombin were lowered to 69.21, 66.76, and 48.84%, respectively. The enzyme substrates are listed in Table 1.

Native human plasmas kallikrein, thrombin, and chymotrypsin were tested for inhibition by r*Ov*SIS (Figure 6). Thrombin was the best inhibited protease at about 50% reduced activity followed by plasmas kallikrein (33%) and chymotrypsin (30%). As only a small number of proteases could be tested, it should just be concluded that *Ov*SIS is an active serpin. Based on the *Ov*SIS sequence TASTTDDQ around the P1 site, low inhibition against these three proteases would be expected, but that would also be the case for trypsin and plasmin. The analysis of this sequence with PeptideCutter [29] (https://web.expasy.org/peptide_cutter/ (accessed on 8 June 2024)) did not result in a hit with any serine protease.

## 4. Discussion

In this investigation, a serpin of the human liver fluke *Opisthorchis viverrini* was characterized with respect to its sequence, structure, phylogeny, and antigenicity. Due to its presence in the infectious stage, it was termed *Ov*SIS, an acronym for ‘**S**erpin **I**nfectious **S**tage’. It represents a new member of the serpin family in the trematodes because it is not orthologous to any of the previously described serpins in this lineage. Based on the phylogenetic analysis shown in Figure 3a, it is in a clade that also contains schistosome SPI, from which it seems to be separated by a few gene duplication events, i.e., it is not an ortholog of SPI. SPI was first isolated and described in *S. haematobium* as an antigenic serpin that was recognized by the sera of schistosome-infected individuals and located on the surface of mature flukes [30]. The antigenicity of *Sh*SPI was confirmed with the finding of specific IgG4 and IgE responses against the protein in infected individuals [31]. Later, the orthologs of *S. japonicum* [32] and *S. mansoni* [33] were described, with comparable findings of tegumental localization and antigenic properties. In this study, *Ov*SIS was found to act as an antigen and to cause a humoral immune response in infected individuals (Figure 5).

In addition to the close phylogenetic relationship and higher sequence conservation, *Ov*SIS shares structural features with SPI, especially the extended helix D [21]. On the other hand, the sequence of the reactive center loop (RCL) around and including the residues that form the reactive bond, which is important for the recognition and interaction with serine proteases, was found to be very different (Figure 3b). The presence of threonine, together with serine at P1 and P2 positions, is unusual, and the observed limited inhibition of chymotrypsin, kallikrein, and thrombin seems to reflect this (Figure 6). Other human serine proteases should be tested in further studies. In addition, the analysis results of schistosome cercarial elastases suggest that the *Ov*SIS sequence from P4 to P4′ TAS**T**TDDQ would not be a preferred substrate of those either [34]. Thus, a specific metacercarial/juvenile serine protease might exist in the genus *Opisthorchis*/*Clonorchis*, which is the native target of *Ov*SIS, and it is possible that the inhibitor acts on certain cysteine proteases as they are also abundant in these flukes in the infectious stage. Serine proteases have not yet been characterized in the genus *Opisthorchis*/*Clonorchis*, and GenBank contains only a small number of predicted membrane-bound and soluble serine protease sequences.

Transcripts of *Ov*SIS were detected in the metacercarial/newly excysted juvenile stages of the parasite but not in 2-week-old juveniles or mature flukes. Therefore, *Ov*SIS must have a time-limited function during the short developmental period, important for the infection process in the human host. Interestingly, *Sm*SPI showed a significant upregulation in 2-h-old schistosomules compared to cercariae and mature flukes [33]. In *S. japonicum*, schistosomules were not tested, but a 1500:1 ratio of *Sj*SPI RNA in mature flukes compared to cercariae was reported [32]. As mentioned in the Introduction Section, unrelated serpins characterized in *C. sinensis* showed varying transcript amounts in different developmental stages too [9,10,11,12]. Differential gene expression during development can occur due to various reasons; it could occur simply because the same target (serine protease) is present at different amounts or that the inhibitor has different/additional functions, for example, as reported in the eggs of mature worms.

Overall, *Ov*SIS is interesting because of its specific presence during the early infection stage, and further investigation of its role during this period could help to better understand the steps of infection and, possibly, how to interfere with this process. It is also important to determine how it stimulates an immune response, i.e., whether it is secreted even without a classical signal peptide and whether it is circulating or attached to the tegument, as observed for schistosome SPI and other trematode serpins. Due to travel restrictions during the recent COVID-19 epidemic, it was not possible to collect sufficient early-stage parasites in this study to perform these analyses. With respect to the biochemical properties of *Ov*SIS, the inhibition profile shows that it could interfere with fibrinolysis as it was found to moderately inhibit thrombin and kallikrein (with low efficiency). However, is that important considering that the juvenile parasite moves quickly and directly into the biliary system and does not feed on blood, even as a mature fluke? Further research is required to identify the protease that *Ov*SIS inhibits in order to better understand its function.

## Figures and Tables

**Table 1:** Final concentrations of enzymes, substrates, and reaction buffers.

Enzyme	Substrate	Buffer
1 nM Chymotrypsin	15 μM Suc-Ala-Ala-Pro-Phe-MCA	100 mM Tris-HCl pH 7.4, 10 mM CaCl_2_
1 nM Kallikrein	30 μM Z-Phe-Arg-MCA	100 mM Tris-HCl pH 7.4, 10 mM CaCl_2_, 0.3 M NaCl
0.1 U Thrombin	30 μM Boc-Val-Pro-Arg-MCA	100 mM Tris-HCl pH 7.4, 10 mM CaCl_2_, 0.3 M NaCl

**Table 2:** Sequence conservation, shown as the percent identity (bold) and similarity (italics) between orthologous serpins of *O. viverrini* (UniProt: A0A075AEP2), *C. sinensis* (UniProt: A0A419PW56), and *O. felineus* (UniProt: A0A4S2MGE0).

Species	*O. viverrini*	*C. sinensis*	*O. felineus*
*O. viverrini*	-----	**83.9**	**82.1**
*C. sinensis*	*90.1*	-----	**87.1**
*O. felineus*	*88.8*	*92.1*	-----

## Data Availability

The nucleotide sequence of *Ov*SIS is available under GenBank accession PP968418. Other data supporting the findings of this study are available from the corresponding author upon request.
